# Can Joint Carbon and Biodiversity Management in Tropical Agroforestry Landscapes Be Optimized?

**DOI:** 10.1371/journal.pone.0047192

**Published:** 2012-10-15

**Authors:** Michael Kessler, Dietrich Hertel, Hermann F. Jungkunst, Jürgen Kluge, Stefan Abrahamczyk, Merijn Bos, Damayanti Buchori, Gerhard Gerold, S. Robbert Gradstein, Stefan Köhler, Christoph Leuschner, Gerald Moser, Ramadhanil Pitopang, Shahabuddin Saleh, Christian H. Schulze, Simone G. Sporn, Ingolf Steffan-Dewenter, Sri S. Tjitrosoedirdjo, Teja Tscharntke

**Affiliations:** 1 Systematic Botany, University of Zurich, Zurich, Switzerland; 2 Plant Ecology, University of Göttingen, Göttingen, Germany; 3 Geoecology/Physical Geography, Institute for Environmental Sciences, University of Koblenz-Landau, Landau, Germany; 4 Faculty of Geography, University of Marburg, Marburg, Germany; 5 Systematic Botany and Mycology, Department of Biology, University of Munich, Munich, Germany; 6 Agroecology, University of Göttingen, Göttingen, Germany; 7 Louis Bolk Institute, LA Driebergen, The Netherlands; 8 Department of Plant Protection, Faculty of Agriculture, IPB, Bogor Agricultural University, Kampus Darmaga, Bogor, Indonesia; 9 Landscape Ecology, Institute of Geography, University of Göttingen, Göttingen, Germany; 10 Muséum National d’Histoire Naturelle, Departement Systématique et Evolution (UMS 602), C.P. 39, Paris, France; 11 Landscape Ecology and Land Evaluation, Faculty for Agricultural and Environmental Sciences, University of Rostock, Rostock, Germany; 12 Department of Plant Ecology, University of Giessen, Giessen, Germany; 13 Department of Biology, Faculty of Mathematics and Natural Sciences, Tadulako University, Palu, Indonesia; 14 Department of Agrotechnology, Faculty of Agriculture, University of Tadulako, Palu, Indonesia; 15 Department of Animal Biodiversity, Faculty of Life Sciences, University of Vienna, Vienna, Austria; 16 FEMS Central Office, CL Delft, The Netherlands; 17 Department of Animal Ecology and Tropical Biology, Biocenter, University of Würzburg, Würzburg, Germany; 18 Department of Biology, Faculty of Mathematics and Natural Sciences, IPB, Bogor Agricultural University, Kampus Darmaga, Bogor, Indonesia; DOE Pacific Northwest National Laboratory, United States of America

## Abstract

Managing ecosystems for carbon storage may also benefit biodiversity conservation, but such a potential ‘win-win’ scenario has not yet been assessed for tropical agroforestry landscapes. We measured above- and below-ground carbon stocks as well as the species richness of four groups of plants and eight of animals on 14 representative plots in Sulawesi, Indonesia, ranging from natural rainforest to cacao agroforests that have replaced former natural forest. The conversion of natural forests with carbon stocks of 227–362 Mg C ha^−1^ to agroforests with 82–211 Mg C ha^−1^ showed no relationships to overall biodiversity but led to a significant loss of forest-related species richness. We conclude that the conservation of the forest-related biodiversity, and to a lesser degree of carbon stocks, mainly depends on the preservation of natural forest habitats. In the three most carbon-rich agroforestry systems, carbon stocks were about 60% of those of natural forest, suggesting that 1.6 ha of optimally managed agroforest can contribute to the conservation of carbon stocks as much as 1 ha of natural forest. However, agroforestry systems had comparatively low biodiversity, and we found no evidence for a tight link between carbon storage and biodiversity. Yet, potential win-win agroforestry management solutions include combining high shade-tree quality which favours biodiversity with cacao-yield adapted shade levels.

## Introduction

Carbon storage in above- and belowground forest vegetation and in the soil plays a crucial role in the terrestrial greenhouse gas balance [1], [2], [3]. After fossil fuel use, tropical deforestation and forest degradation represent the second largest source of carbon emissions, contributing about 12–20% of the annually released CO_2_ [4], [5]. Accordingly, conservation of tropical forests and reforestation of formerly forested habitats are viewed as important components of global strategies to reduce CO_2_ emissions [6]. At the same time, tropical forests harbour some of the highest levels of biodiversity on Earth as well as the largest number of species threatened with global extinction [7]. This dual role of tropical forests as carbon and biodiversity repositories presents a potential win-win situation, in which management of habitats for carbon storage may in parallel result in biodiversity conservation [8], [9], [10]. Over the last decade, there have been several moves towards establishing payment schemes, in which tropical forest conservation or reforestation initiatives are remunerated [11]. Politically, such proposals are hotly debated, as both the controlling mechanisms and the potential benefits are unclear [12], [13], [14].

There is little doubt that the preservation of large tracts of natural tropical forests will safeguard both large carbon stocks and the habitats of much threatened fauna and flora, especially for those species dependent on undisturbed habitats and with low population densities, therefore requiring large habitat tracts to persist [15], [16]. However, human-impacted ecosystems, including logged natural forest and secondary forests as well as agricultural areas, cover ever-increasing areas and play a crucial role both in carbon and biodiversity management [17], [18]. Indeed, several of the proposed carbon payment schemes exclusively focus on the management of impacted ecosystems as carbon sinks [19]. The Kyoto Protocol, for example, explicitly excluded the reduction of emissions by avoiding deforestation because of both political and technical obstacles.

In agricultural systems, the relationship of carbon stocks and biodiversity is far from clear [16], [20], [21]. Globally, 46% of the agricultural area has at least 10% tree cover, and can thus be classified as agroforests [22]. Amongst them, agroforests holding a substantial tree cover (at least 30%) still account for as much as 374 million hectares [22]. In 2007, agroforests for coffee and cacao production in tropical landscapes, which are the second- and third-largest international trade commodities after petroleum, covered no less than 17.7 million hectares worldwide [23]. Because agroforests are tree-dominated ecosystems, they potentially play an important role for carbon management [24]. At the same time, agroforests can harbour significant levels of biodiversity [25], [26], [27]. Yet, the potential links between carbon stocks and biodiversity levels in tropical agroforests as a basis for environmentally optimized agroforestry management remain unexplored.

In the present study, we assessed the potential to optimize carbon management in agroforests while at the same time safeguarding high levels of biodiversity. Because natural forest-based biodiversity is commonly considered to be the most threatened in tropical forest ecosystems [5], [28], we placed a special focus on the richness of species that we recorded in the natural forest habitats.

## Materials and Methods

Our study complies with the current laws of Indonesia and Germany and with international rules. Permissions for fieldwork in Indonesia and collecting and exporting samples have been provided by national and local authorities.

### Study Area and Site Selection

The study took place around the village of Toro (1°30′24′′ S, 120°2′11′′ E) located at the western border of Lore Lindu National Park, about 100 km south of Palu, the capital city of Central Sulawesi, Indonesia. The natural vegetation around the village is submontane rainforest. The agricultural landscape in the region consists of pastures, paddy fields and cacao-dominated agroforests. Cacao production in the region increased strongly in the 1990s. The cacao agroforests are managed by small-scale farmers. Shade tree management in the region is dynamic and farmers tend to remove shade trees in mature agroforestry systems to increase cacao production [26].

We defined four habitat types with different shade tree diversity [29]: (1) natural forest sites, situated at least 300 m away from forest sites where selective logging occurred; (2) cacao agroforests with diverse, natural shade trees, retained after thinning of the previous forest cover, underplanted with cacao trees and few fruit trees (high shade agroforests); (3) cacao agroforests with shade tree stands dominated by various species of planted fruit and timber trees (medium shade); (4) cacao agroforests with a low diversity of planted shade trees, predominantly non-indigenous, nitrogen-fixing leguminous trees and a few native fruit tree species (low shade).

We randomly selected 3–4 replicates from a larger subset of each habitat type. Natural forest sites were chosen that were representative for rain forests in this region and elevation belt in terms of forest structure and tree species composition. Agroforest sites were selected based on the age of the cacao trees, which was at all sites 4–17 years. At the time of this study, farmers regularly pruned trees and weeded the plantations and only rarely treated them with fertilizers and pesticides.

Distance between study sites ranged between 0.3–5 km. All sites were at 850–1100 m above sea level. The agroforests did not have sharp borders, but gradually changed into other forms of land-use and at the landscape scale formed a continuous band along the forest margin. We marked core areas of 50×50 m^2^ in the middle of each site, whose land-use and shade tree composition was as constant as possible. Sites belonging to the different habitat types were not spatially clustered, but geographically interspersed.

As for all observational studies regarding natural forest conversion, our comparative study approach could have been affected by confounding effects such as that the chosen natural forest plots were not typical for the area and therefore not representative for those forest stands that have been converted prior to the recent farmer agroforest management or that farmers preferentially converted low-biodiversity forest for some reasons. However, recent conversion of former natural forest into agroforestry systems is still common in the study area, and there are no apparent biases in where clearing takes place (except that more accessible and less steep sites are preferred, but this was taken into account by our sampling design). This together with the fact that the natural forest structure and composition is quite homogenous in the entire study area (rather species-rich with comparable carbon stocks) makes it unlikely that our results have been affected by such confounding effects.

### Carbon Stock Estimation in Above- and Belowground Tree Biomass

The estimation of above-ground tree biomass of the natural forest trees was conducted according to the methods described in [30]. The procedure followed common standard procedures and is based on stand inventories of above-ground tree dimensions, data on wood-specific density of the present genera, and the application of allometric equation models from the literature [e.g. 31,32]. The below-ground biomass of all forest trees in each plot was estimated using the root/shoot ratio from [33] for tropical-subtropical moist forest and plantations. For trees in plots with an above-ground biomass (AGB) <125 Mg ha^−1^, the applied root:shoot ratio was 0.205, while for trees from plots with AGB >125 Mg ha^−1^ it was 0.235. Above- and belowground biomass of cacao and planted *Gliricidia* trees were estimated from stem diameter records using allometric relationships for above-ground biomass as well as root/shoot ratios established in the nearby plots [34]. The root/shoot ratios in this study were 0.394 for *Theobroma* and 0.488 for *Gliricidia*. For the calculation of the above- and below-ground biomass of *Coffea* trees, we used the allometric equation from [35].

The above- and below-ground biomass sums of all inclined plots were transformed to the horizontal projection and are given as Mg ha^−1^. Carbon stock sizes were calculated from the biomass data applying data on carbon contents of 42% for above-ground and 46% for below-ground biomass that were measured in nearby forest plots [30].

### Soil Carbon Stock Estimation

We sampled each plot at least six times and excavated representative soil pits. Soils were sampled per horizon until the depth of 1 m. Soil analyses were performed on the fine earth fraction (<2 mm). Stone contents (vol%) were estimated in the field. Bulk densities were measured using undisturbed soil cores (100 cm^3^) after drying at 105°C. The carbon content (g carbon per kg soil) was determined for each horizon by analyzing air dried soil samples with a Vario El CN Analyser (Elementar, Hanau, Germany) in the laboratory in Palu, Indonesia. For each horizon the carbon stock (C_stock_, in Mg ha^−1^) was calculated using the equation C_stock_ = C_conc_ × BD × d × a × CF_stones_ following [36], where C_conc_ represents the carbon content (in g kg^−1^), BD is the soil bulk density of the respective horizon (in kg m^−3^), d is the thickness of the horizon (in m), the related plot area a (one ha = 10,000 m^2^), and a correction factor for the stone content of the soil samples (CFstones, (100–%stones)/100). The calculated soil carbon stocks per horizon were summed up to one meter depth and given as Mg ha^−1^ for each plot.

### Quantification of Biodiversity

We assessed the species richness of trees, lianas and herbs, epiphytic liverworts from lower canopy trees, birds, butterflies, ants and beetles from lower canopy trees, dung beetles, bees, wasps, and their parasitoids for each 50×50 m^2^ plot according to the methods described in [29] and briefly summarized here. In the largely aseasonal climate of our study region (mean monthly precipitation is >100 mm for each month; mean monthly temperatures vary <2°C over the year) no marked seasonal variations of species composition and abundance were detected over several years of field work [e.g. 26,37]. Accordingly, timing of the sampling should not have influenced main patterns of our results. *Trees:* All trees dbh ≥10 cm were mapped and individually numbered with aluminum tags, their dbh was measured, and their trunk height and total height were estimated. *Lianas and herbs:* In each study plot of 50×50 m^2^ ten subplots of 2×2 m^2^ each were randomly placed. Within these, all herb and liana species were inventoried, collected, and determined. *Epiphytic liverworts from lower canopy trees:* Two trees with a height up to 8 m, a dbh ranging of 20–60 cm, and comparable bark texture were selected in each study plot. Each tree was divided into zone 1 (treebase up to the first ramification), zone 2 (inner crown) and zone 3 (outer crown) according to modified Johansson zones for small trees. Within subplots of 200 cm^2^, liverworts were sampled from each cardinal direction in all three zones. *Birds:* Each plot was visited on two mornings from 05∶30 to 10∶30 am. Birds were recorded visually and acoustically, and by systematic tape recordings. *Butterflies:* Butterflies were captured alive in traps baited with rotten mashed bananas in traps suspended from tree branches with strings about 1.5 m above the ground. *Ants and beetles from lower canopy trees:* Within each study plot, four trees were selected, which were of similar age and size. The insect fauna was sampled using canopy knockdown fogging, using a SwingFog TF35 to blow a fog of 1% pyrethroid insecticide (Permethrin) Killed arthropods were collected from a 4 m^2^ sheet of white canvas placed directly under each tree. *Dung beetles:* Dung beetles were collected using baited pitfall traps baited with ca. 20 g of fresh cattle (*Bos taurus*) dung. *Bees, wasps, and their parasitoids:* Trap nests offer standardized nesting sites for above-ground nesting bees and wasps and can therefore be used to experimentally study these insects. They were constructed from PVC tubes with a length of 28 cm and a diameter of 14 cm. Internodes of the reed *Saccharum spontaneum* with varying diameter (3–25 mm) and a length of 20 cm were inserted into these tubes to provide nesting sites. Twelve trap nests (four in each stratum) were installed in three different heights from understorey and intermediate tree height to the canopy. Trap nests were checked every month and bee and wasp larvae were reared for later identification.

### Correlations of Carbon Stocks and Biodiversity

To arrive at a comprehensive measure of biodiversity for each plot, we combined all groups. In order to weight all groups similarly, we first standardized the richness values of each group by setting the maximum plot count at 100% and all other counts respectively. We then averaged the standardized values for all 12 groups for each plot. This approach has previously been used to obtain generalized biodiversity patterns when numerous taxa have been sampled [38], [39]. We then calculated simple, linear determination values (R^2^) to relate carbon stocks to biodiversity. This was done for the entire carbon stocks as well as separately for above- and below-ground (soil + root) carbon stocks. Because relationships may be driven by the marked contrasts between natural forests and agroforests, both for biodiversity [29] and carbon stocks [40], in a second step we only included the 11 agroforestry plots in the analyses. Finally, because forest-based biodiversity is considered to be the most threatened in tropical forest ecosystems [5], [28], we repeated all analyses by only including those species recorded in the natural forests of the study region [29]. In order to also be able to assess group-specific patterns, we repeated the above analyses for all groups independently (Figures S1, S2, S3). All statistical analyses have been done using the statistical platform R [41].

## Results

**Figure 1:**
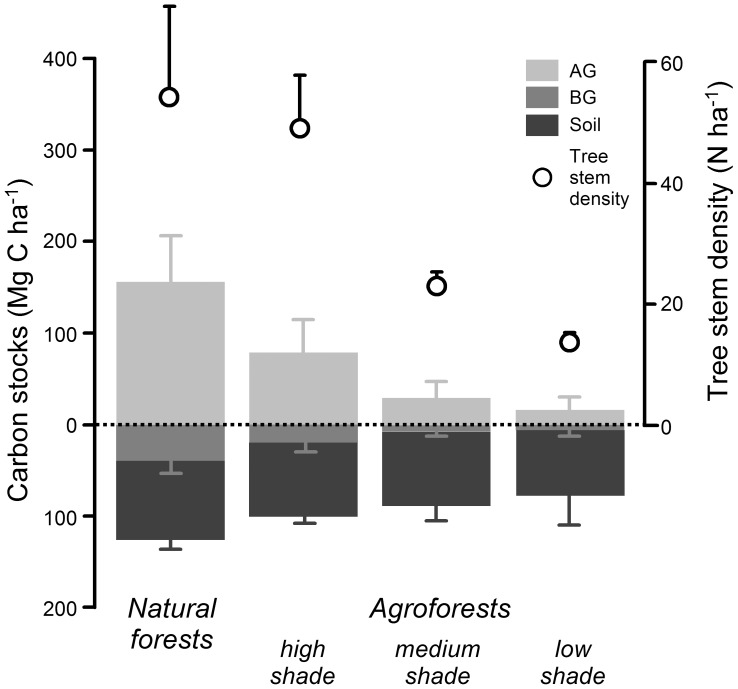
Carbon stocks in natural rainforests and cacao agroforests of varying tree density and shade levels. Shade levels were defined as: high shade: cacao agroforests with diverse, natural shade trees, retained after thinning of the previous forest cover, underplanted with cacao trees and few fruit trees; medium shade: cacao agroforests with shade tree stands dominated by various species of planted fruit and timber trees; low shade: cacao agroforests with a low diversity of planted shade trees, predominantly non-indigenous, nitrogen-fixing leguminous trees and a few native fruit tree species. Columns show mean carbon stocks (+1 SD) in the above-ground (AG) and below-ground (BG) plant components as well as in the soil. Also shown is the mean stem density (+1 SD) of trees with diameters ≥10 cm at breast height.

In natural forests, carbon stocks were on average over twice as high as in the agroforests (Figure 1). The above-ground vegetation in natural forests held on average 54% of the total carbon stocks, with the root and soil components each contributing 14% and 32%, respectively. In agroforests, in contrast, the soil component on average included about 66% of the carbon stocks, followed by the above-ground vegetation (26%) and roots (7%). Perhaps the most striking result was that soil carbon stocks did not differ significantly between natural forests and agroforests (t-test, t = 0.68, P = 0.51), on average declining only from 87 Mg C ha^−1^ to 80 Mg C ha^−1^, although a single agroforest plot had a value of 40 Mg C ha^−1^.

**Figure 2:**
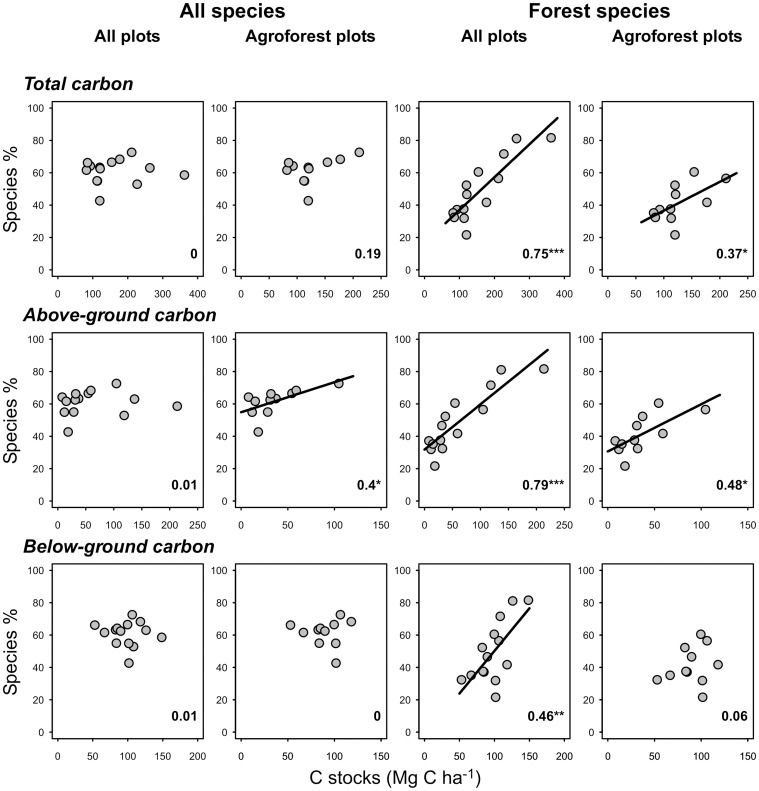
Relationships of species richness to carbon stocks. Relationships of species richness to carbon stocks, separated for all species and only those species recorded in the natural forest (forest species), for total, above-ground and below-ground carbon stocks as well as for all 14 study plots and only the 11 agroforest plots. To summarize the species richness patterns of the 12 focal plant and animal groups, richness values were all standardized to 100% relative to the highest plot values of each group and then averaged across all taxa. All individual relationships are shown in Figs. S1–3. Numbers in each graph are coefficients of determination (R^2^-values), trend lines are shown for significant relationships only. *p<0.05, **p<0.01, ***p<0.001.

The relationship of the species richness of all species to carbon stocks showed no or only marginally significant patterns, both when all plots and only the agroforest plots were considered (Figure 2, Figures S1, S2, S3). In contrast, when we only considered the forest-related species, we obtained highly significant relationships between species richness and carbon stocks when all plots were included. When we restricted this analysis to the agroforest plots, relationships were weaker but still significant for total and above-ground carbon stocks.

When we analyzed the species groups individually, only 1–3 groups showed significant positive or negative relationships to total carbon stocks, particularly trees, bryophytes and dung beetles (positive) as well as herbs, wasps, and their parasitoids (negative) (Figures S1, S2, S3). When we restricted the same analyses to the agroforestry plots, only a single relationship (lianas) was significant. When we restricted the analysis to forest species, no less than 23 of 36 (64%) of the relationships were significant. When the forest plots were excluded, median r-values decreased to 0.4–0.5 and only 8 (22%) relationships remained significant. In all cases except the analyses with all species across all plots, R^2^ values were slightly higher when considering above-ground carbon stocks than below-ground stocks, with overall values intermediate.

Because trees are directly managed by the local farmers aiming to manipulate the shading level of cacao plantations, we explored the relationships of trees and biodiversity in more detail. Tree species richness was significantly positively correlated to the species richness of only four of the other eleven study groups (Table 1). However, when we only considered the richness of tree species from the natural forests, the regression values between tree species richness and richness of the other groups were significantly higher, both across all study plots and in the agroforest plots only.

**Table 1:** Results of Wilcoxon’s tests for matched pairs, comparing (a) the linear correlation (r-)values of the species richness of various study groups against tree species richness with (b) the r-values of the species richness of the same study groups against the species richness of natural forest trees, either in all study plots (upper half of the table) or only in the agroforest study plots (lower half).

	All study groups(N = 11)		Only animal groups(N = 8)	
	W	P	W	P
All plots (N = 14)				
All species	22	n.s.	12	n.s.
Forest-adapted species	10	<0.05	7	<0.05
Only agroforest plots (N = 11)				
All species	31	n.s.	14	n.s.
Forest-adapted species	11	<0.05	0	<0.01

W  =  test statistic, P  =  probability.

## Discussion

Overall, we found that the relationship between carbon stocks and biodiversity was fairly weak and most pronounced when considering only forest-based biodiversity as well as when contrasting forest plots with agroforest plots. One may argue that the fact that carbon storage in the above-ground biomass of the agroforestry systems (being less bio-diverse) is less than that in the natural forest (harbouring higher biodiversity) are to be expected due to the large differences in stand structure. However, we also found that there is no simple, linear relationship between forest structure and biodiversity. While the simplistic assumption that more tree biomass automatically leads to higher biodiversity only holds true when we contrast natural forest with agroforestry systems, no such simple relationship is evident in different types of the latter. We therefore conclude that the conservation of carbon stocks and in particular of the forest-related biodiversity mainly depends on the preservation of natural forest habitats. Reduction of canopy tree density and cover within agroforestry systems leads to substantial carbon losses of around 50 Mg C ha^−1^, but only to limited and taxon-specific biodiversity losses. Our study thus suggests that remuneration schemes aimed at preserving or increasing carbon stocks in tropical forest regions should focus on maintaining natural forest ecosystems, in support of current political initiatives for Reducing Emissions from Deforestation and forest Degradation (REDD) [12], [13], [14].

On the other hand, carbon storage of the three most carbon-rich agroforest systems was about 60% of that of the three natural forest plots (181 *versus* 284 Mg C ha^−1^), suggesting that 1.6 ha of optimally managed agroforest could contribute to the conservation of carbon stocks as much as 1 ha of natural forest. As for all observational studies, this result is only valid if the studied natural forest plots were typical for the whole study area and therefore representative for those forest stands that have been converted prior to the recent farmer agroforest management, which was the case in our study (Steffan-Dewenter et al. 2007). In particular, we found that land use change towards agroforests did not lead to excessive losses of the long-term soil carbon stocks, unlike observed in ploughed arable land-use systems, where up to 25–30% of the soil carbon is commonly lost in the tropics [40]. Agroforests may, in terms of soil organic carbon, thus be closer to secondary forests, which on average have 9% less soil carbon than primary forests [40]. We suspect that the reason for the limited loss of carbon stocks in the soil is that in our study region the creation of agroforests usually is not achieved via total removal of tree cover that leads to strong erosion and decomposition, but rather through the partial removal of natural trees and gradual replacement by other tree species [26], thus preserving much of the root systems and preventing erosion and decomposition. From the point of view of carbon stock management, such a gradual transition is therefore preferable to the wholesale removal of natural tree cover and replacement by agroforest trees. However, since the carbon flux to the soil via leaf and root litter is markedly lower in agroforestry systems than in the natural forest [42], the soil carbon stocks might become lower in the long run.

In agroforests, we failed to detect a close relationship between carbon stocks and the species richness of most taxa. Thus, management strategies to maximise carbon storage, as supported by the Kyoto Protocol, do not automatically enhance biodiversity in agroforests. This raises the need to identify promising solutions to optimise both carbon and biodiversity management in these economically and ecologically important agricultural systems. We explored this option by contrasting the carbon-biodiversity relationships when considering native versus non-native trees and found that biodiversity was more closely linked to the former. This suggests that carbon-biodiversity win-win solutions can be achieved when not all natural trees are removed during forest conversion. This may be further optimized by focussing on the identity of the tree species through shade-tree management that combines shade levels allowing for both high yield and low cacao stress with a selection of diverse shade-tree species from natural forests that enhance the biodiversity of other taxa [43]. This, and possibly other similar relationships involving functional traits of the shade trees such as fruit quantity and quality, opens promising perspectives for optimised joint carbon-biodiversity management strategies in agroforests.

## Supporting Information

Figure S1 **Species richness of selected organisms in relation to total carbon stocks in the 14 plots.** Species richness (number of species per plot) of 12 groups of organisms in relation to total carbon stocks in 14 plots of natural forest and cacao agroforests. Large black circles denote natural forest plots, small circles agroforests of varying tree density (white: 0–79 trees >20 cm dbh/ha; medium: 80–159 trees/ha; dark: 160–240 trees/ha) with blue symbols showing total species richness and red symbols richness of species also recorded in the natural forest. Coefficients of determination values (R^2^ values) including all plots are given in normal font and for significant relationships are illustrated by continuous lines, values only including the agroforests are given in italics and illustrated by dashed lines. *p<0.05, **p<0.01, ***p<0.001.
(TIF)

Figure S2 **Species richness in the study plots in relation to below-ground carbon stocks.** Species richness of 12 groups of organisms in relation to below-ground (soil + root) carbon stocks in 14 plots of natural forest and cacao agroforests. Symbols as in Fig. S1.
(TIF)

Figure S3 **Species richness in the study plots in relation to above-ground carbon stocks.** Species richness of 12 groups of organisms in relation to above-ground carbon stocks in 14 plots of natural forest and cacao agroforests. Symbols as in Fig. S1.
(TIF)
