# Supplementary figures and images for: Can Joint Carbon and Biodiversity Management in Tropical Agroforestry Landscapes Be Optimized?
Source: PLoS One. 2012 Oct 15;7(10):e47192. doi: 10.1371/journal.pone.0047192 (PMC3471943; doi:10.1371/journal.pone.0047192)

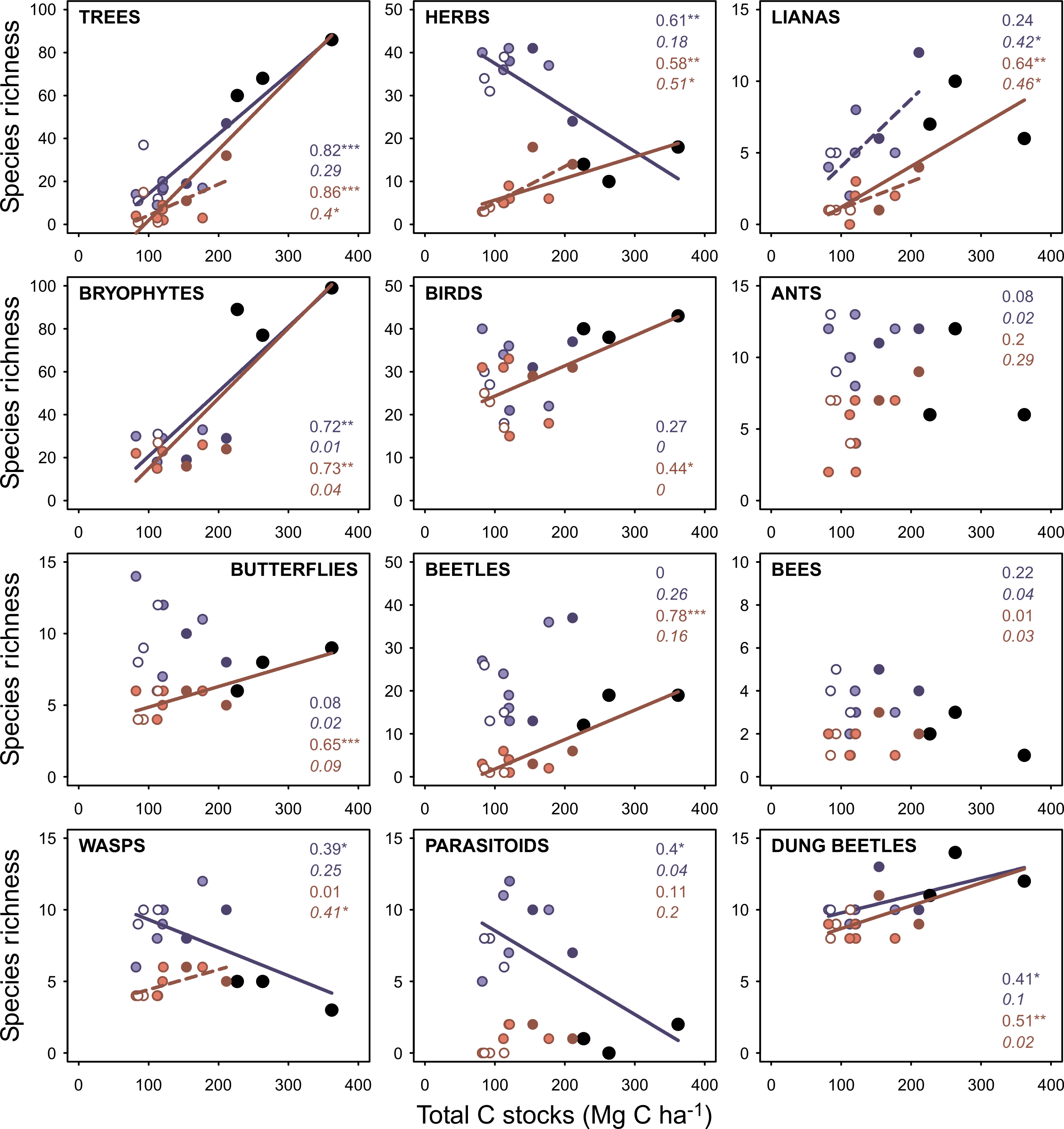

Supplement: Figure S1 — Species richness of selected organisms in relation to total carbon stocks in the 14 plots. Species richness (number of species per plot) of 12 groups of organisms in relation to total carbon stocks in 14 plots of natural forest and cacao agroforests. Large black circles denote natural forest plots, small circles agroforests of varying tree density (white: 0–79 trees >20 cm dbh/ha; medium: 80–159 trees/ha; dark: 160–240 trees/ha) with blue symbols showing total species richness and red symbols richness of species also recorded in the natural forest. Coefficients of determination values (R2 values) including all plots are given in normal font and for significant relationships are illustrated by continuous lines, values only including the agroforests are given in italics and illustrated by dashed lines. *p<0.05, **p<0.01, ***p<0.001. (TIF) [file pone.0047192.s001.tif]

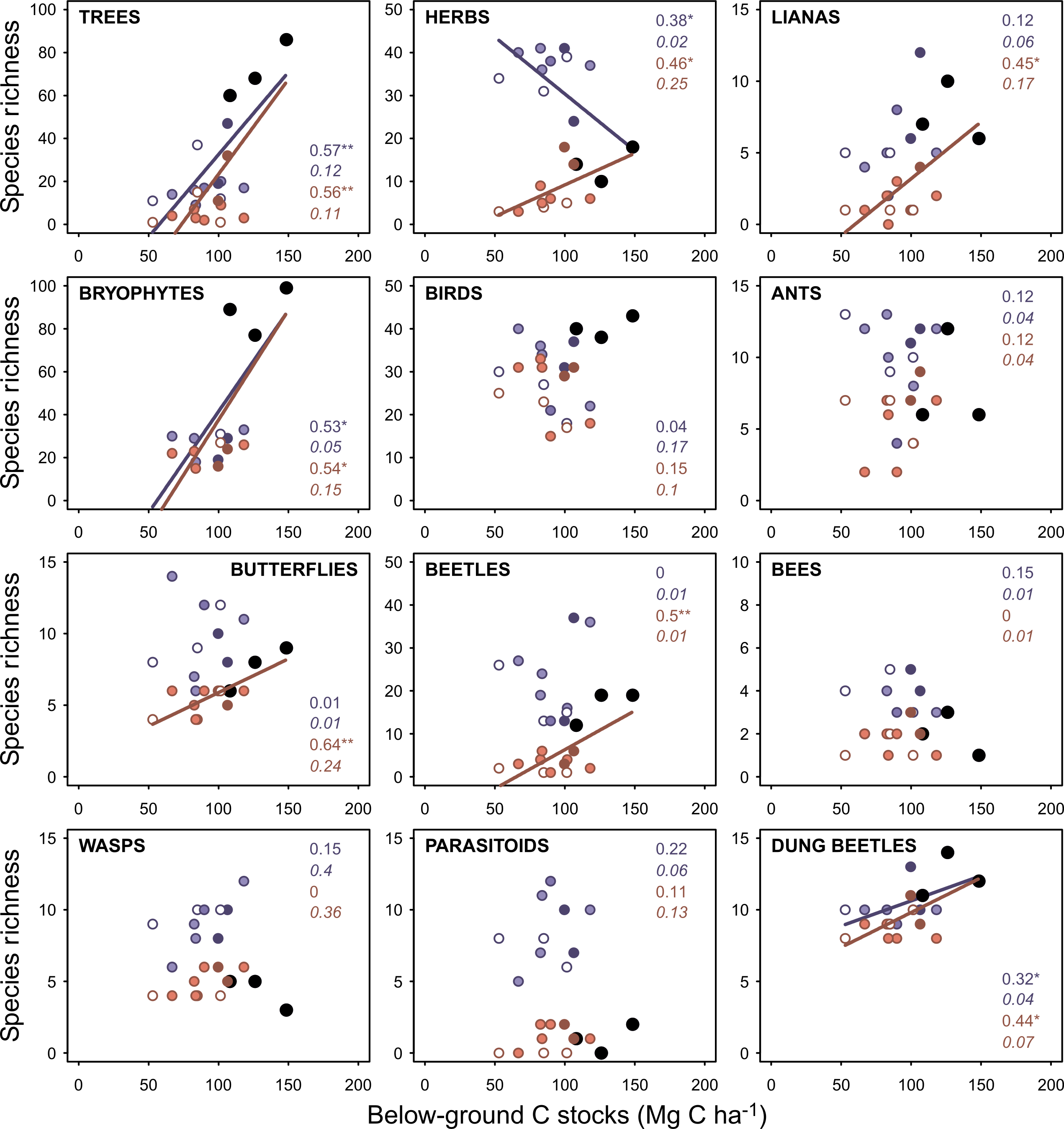

Supplement: Figure S2 — Species richness in the study plots in relation to below-ground carbon stocks. Species richness of 12 groups of organisms in relation to below-ground (soil + root) carbon stocks in 14 plots of natural forest and cacao agroforests. Symbols as in Fig. S1. (TIF) [file pone.0047192.s002.tif]

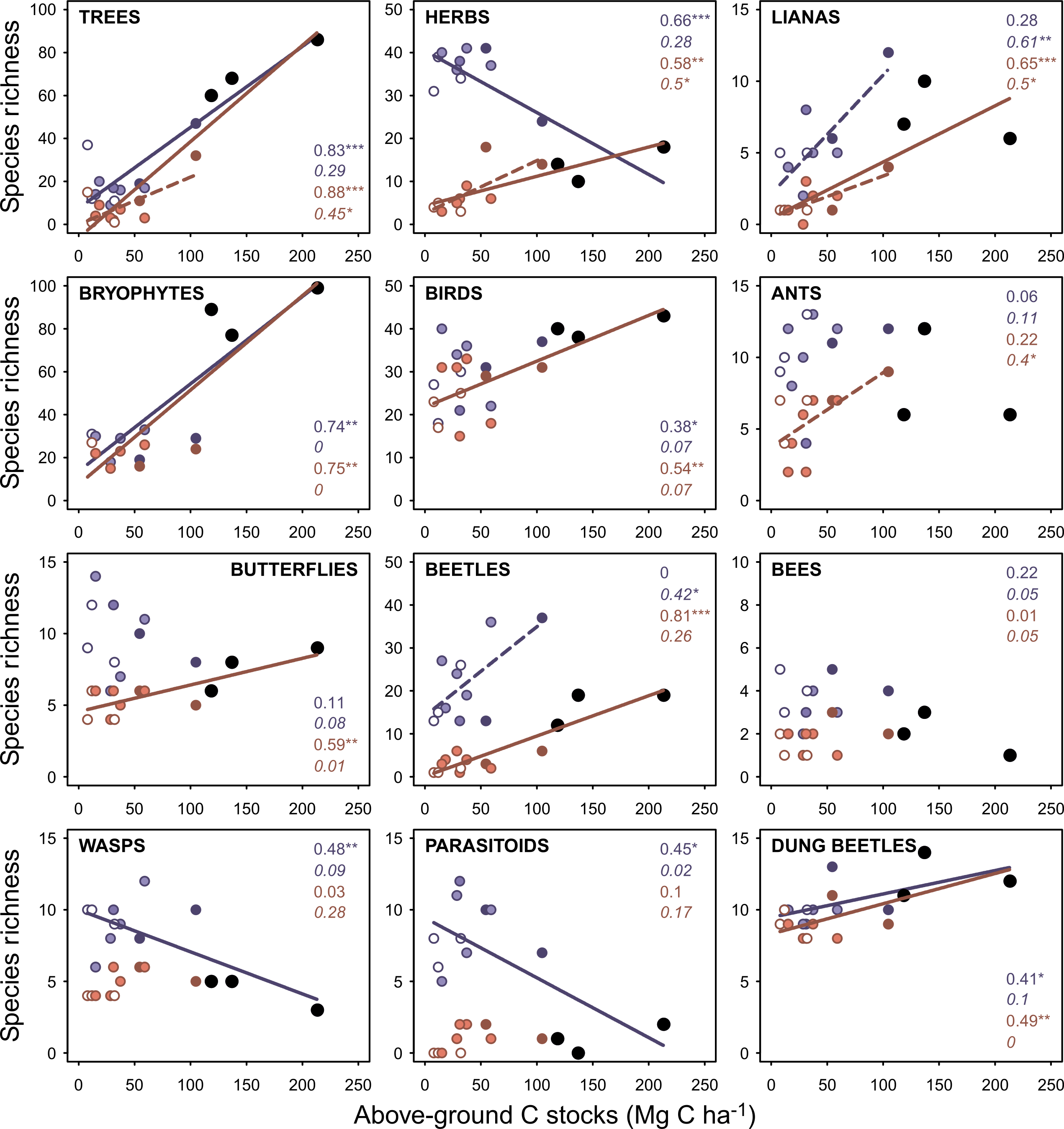

Supplement: Figure S3 — Species richness in the study plots in relation to above-ground carbon stocks. Species richness of 12 groups of organisms in relation to above-ground carbon stocks in 14 plots of natural forest and cacao agroforests. Symbols as in Fig. S1. (TIF) [file pone.0047192.s003.tif]
